# 1,1′-(2-Thienylmethylene)di-2-naphthol ethyl acetate solvate

**DOI:** 10.1107/S1600536809016559

**Published:** 2009-05-14

**Authors:** Yuan Zhang, Yong Hua Li, Min Min Zhao, De Hong Wu, Rong Yang

**Affiliations:** a Ordered Matter Science Research Center, College of Chemistry and Chemical Engineering, Southeast University, Nanjing 211189, People’s Republic of China

## Abstract

In the title compound, C_25_H_18_O_2_S·C_4_H_8_O_2_, there are inter­molecular O—H⋯O hydrogen bonds between the main mol­ecule and the solvent molecule. The thio­phene ring is oriented at dihedral angles of 70.87 (7) and 75.36 (4)° with respect to the mean planes of the two naphthyl ring systems.

## Related literature

For the properties of bis­naphthols, see: Handique & Barauh *et al.* (2002 ▶). For bond-length data, see: Allen *et al.* (1987 ▶).
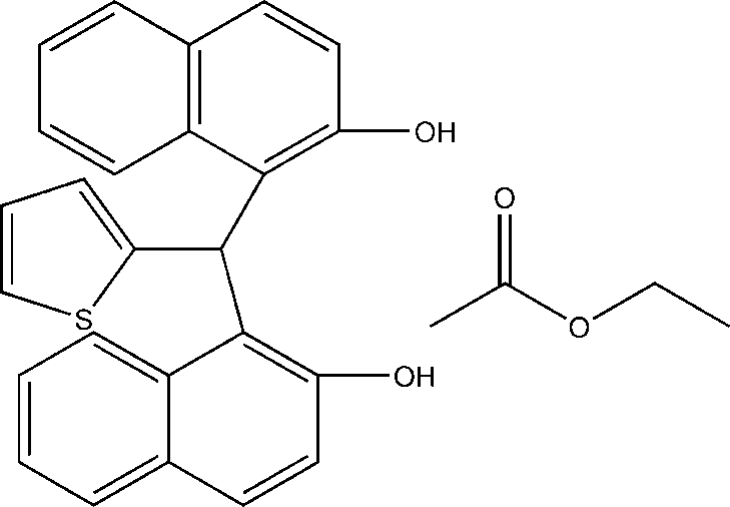


## Experimental

### 

#### Crystal data

C_25_H_18_O_2_S·C_4_H_8_O_2_
*M* *_r_* = 470.57
Monoclinic, 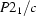

*a* = 13.425 (3) Å
*b* = 21.613 (4) Å
*c* = 8.417 (2) Å
β = 98.808 (15)°
*V* = 2413.4 (9) Å^3^
*Z* = 4
Mo *K*α radiation
μ = 0.17 mm^−1^
*T* = 291 K
0.40 × 0.27 × 0.25 mm

#### Data collection

Rigaku SCXmini diffractometer
Absorption correction: multi-scan (*SADABS*; Bruker, 2000 ▶) *T*_min_ = 0.95, *T*_max_ = 0.96
23800 measured reflections
5314 independent reflections
4010 reflections with *I* > 2σ(*I*)
*R*_int_ = 0.047

#### Refinement

*R*[*F* ^2^ > 2σ(*F* ^2^)] = 0.063
*wR*(*F* ^2^) = 0.158
*S* = 1.00
5314 reflections
309 parameters
H-atom parameters constrained
Δρ_max_ = 0.19 e Å^−3^
Δρ_min_ = −0.31 e Å^−3^

### 

Data collection: *CrystalClear* (Rigaku, 2005 ▶); cell refinement: *CrystalClear*; data reduction: *CrystalClear*; program(s) used to solve structure: *SHELXS97* (Sheldrick, 2008 ▶); program(s) used to refine structure: *SHELXL97* (Sheldrick, 2008 ▶); molecular graphics: *SHELXTL/PC* (Sheldrick, 2008 ▶); software used to prepare material for publication: *SHELXTL/PC*.

## Supplementary Material

Crystal structure: contains datablocks I, global. DOI: 10.1107/S1600536809016559/gw2064sup1.cif

Structure factors: contains datablocks I. DOI: 10.1107/S1600536809016559/gw2064Isup2.hkl

Additional supplementary materials:  crystallographic information; 3D view; checkCIF report

## Figures and Tables

**Table 1:** Hydrogen-bond geometry (Å, °)

*D*—H⋯*A*	*D*—H	H⋯*A*	*D*⋯*A*	*D*—H⋯*A*
O2—H2*A*⋯O3^i^	0.91	1.88	2.764 (3)	163

Symmetry code: (i) 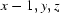.

**Fig. 1.**
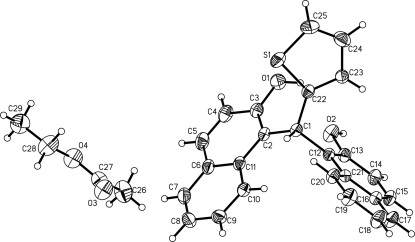
Perspective structure of the title compound, showing the atom-numbering scheme. Displacement ellipsoids are drawn at the 30% probability level.

**Fig. 2.**
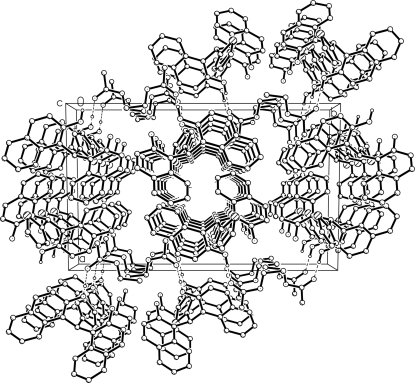
The crystal packing of the title compound viewed along the c axis showing hydrogen bondings network.

## supplementary crystallographic information

### Comment

The molten reaction of 2-naphthol, thiophene-2-carbaldehyde and 1-*p*-tolylethanamine at 120°C did not yield a Betti-type product, but the title bisnaphthol compound. Bisnaphthols are usually referred to as a diverse group of synthetic compounds containing two naphthol units which are connected by an aldehyde group. They have synthetic, medicinal and industrial value (Handique & Barauh *et al.* 2002). Here we report the synthesis and crystal structure of the title compound. The asymmetric unit of the compound contains an ethyl acetate solvent molecule (Fig. 1). The bond lengths and angles are within normal ranges (Allen *et al.* 1987).

Rings of the two naphthols and thiophene are, of course, planar. The dihedral angles between rings A (C2–C6/C11) and B (C6–C11), and between rings C (C12–C16/C21) and D (C16–C21), are 0.87 (4) and 1.57 (3), respectively. The orientation of ring E (C22–C25/S1) with respect to the mean planes of the two naphthyl groups containing rings A and B, and C and D, may be described by the dihedral angles of 70.87 (7) and 75.36 (4), respectively. The dihedral angle between the mean planes of the two naphthyl groups is 75.36 (4).

As can be seen from the packing diagram (Fig. 2), intermolecular O—H···O hydrogen bonds (Table 1) link the molecules. Dipole–dipole and van der Waals interactions are also effective in the molecular packing.

### Experimental

Thiophene-2-carbaldehyde (1.68 g, 0.015 mol) and 1-*p*-tolylethanamine (2.025 g, 0.015 mol) was added to 2-naphthol (2.16 g, 0.015 mol) without solvent under nitrogen. The temperature was raised to 120°C in one hour gradually and the mixture was stirred at this temperature for 10 h. The system was treated with 20 ml of ethanol 95% and cooled. The precipitate was filtered and washed with a small amount of ethanol 95%. The title compound was isolated using column chromatography (petroleum ether:ethyl acetate 2:1). Single crystals suitable for X-ray diffraction analysis were obtained from slow evaporation of ethyl acetate solution.

### Refinement

H atoms were positioned geometrically and refined using a riding model, with C—H = 0.91–0.96 Å and *U*_iso_(H) = 1.3–1.5*U*_eq_(C).

### Crystal data

**Table:**

C_25_H_18_O_2_S·C_4_H_8_O_2_	*F*(000) = 992
*M**_r_* = 470.57	*D*_x_ = 1.295 Mg m^−^^3^
Monoclinic, *P*2_1_/*c*	Mo *K*α radiation, λ = 0.71073 Å
Hall symbol: -P 2ybc	Cell parameters from 4944 reflections
*a* = 13.425 (3) Å	θ = 2.4–27.2°
*b* = 21.613 (4) Å	µ = 0.17 mm^−^^1^
*c* = 8.417 (2) Å	*T* = 291 K
β = 98.808 (15)°	Prism, colourless
*V* = 2413.4 (9) Å^3^	0.40 × 0.27 × 0.25 mm
*Z* = 4	

### Data collection

**Table:**

Rigaku SCXmini diffractometer	5314 independent reflections
Radiation source: fine-focus sealed tube	4010 reflections with *I* > 2σ(*I*)
graphite	*R*_int_ = 0.047
Detector resolution: 13.6612 pixels mm^-1^	θ_max_ = 27.1°, θ_min_ = 3.1°
CCD Profile fitting scans	*h* = −17→17
Absorption correction: multi-scan (*SADABS*; Bruker, 2000)	*k* = −27→27
*T*_min_ = 0.95, *T*_max_ = 0.96	*l* = −10→10
23800 measured reflections	

### Refinement

**Table:**

Refinement on *F*^2^	Primary atom site location: structure-invariant direct methods
Least-squares matrix: full	Secondary atom site location: difference Fourier map
*R*[*F*^2^ > 2σ(*F*^2^)] = 0.063	Hydrogen site location: inferred from neighbouring sites
*wR*(*F*^2^) = 0.158	H-atom parameters constrained
*S* = 1.00	*w* = 1/[σ^2^(*F*_o_^2^) + (0.0651*P*)^2^ + 1.3572*P*] where *P* = (*F*_o_^2^ + 2*F*_c_^2^)/3
5314 reflections	(Δ/σ)_max_ < 0.001
309 parameters	Δρ_max_ = 0.19 e Å^−^^3^
0 restraints	Δρ_min_ = −0.31 e Å^−^^3^

### Special details

**Table:**

Geometry. All e.s.d.'s (except the e.s.d. in the dihedral angle between two l.s. planes) are estimated using the full covariance matrix. The cell e.s.d.'s are taken into account individually in the estimation of e.s.d.'s in distances, angles and torsion angles; correlations between e.s.d.'s in cell parameters are only used when they are defined by crystal symmetry. An approximate (isotropic) treatment of cell e.s.d.'s is used for estimating e.s.d.'s involving l.s. planes.
Refinement. Refinement of *F*^2^ against ALL reflections. The weighted *R*-factor *wR* and goodness of fit *S* are based on *F*^2^, conventional *R*-factors *R* are based on *F*, with *F* set to zero for negative *F*^2^. The threshold expression of *F*^2^ > σ(*F*^2^) is used only for calculating *R*-factors(gt) *etc*. and is not relevant to the choice of reflections for refinement. *R*-factors based on *F*^2^ are statistically about twice as large as those based on *F*, and *R*- factors based on ALL data will be even larger.

### Fractional atomic coordinates and isotropic or equivalent isotropic displacement parameters (Å^2^)

**Table:**

	*x*	*y*	*z*	*U*_iso_*/*U*_eq_	
C1	0.31470 (16)	0.04909 (9)	0.4961 (3)	0.0371 (5)	
H1	0.3802	0.0292	0.4957	0.045*	
C2	0.33844 (16)	0.10811 (9)	0.5991 (3)	0.0376 (5)	
C3	0.28873 (17)	0.16412 (10)	0.5686 (3)	0.0451 (5)	
C4	0.3180 (2)	0.21791 (11)	0.6593 (3)	0.0573 (7)	
H4	0.2841	0.2550	0.6336	0.069*	
C5	0.3952 (2)	0.21569 (12)	0.7836 (3)	0.0591 (7)	
H5	0.4135	0.2513	0.8429	0.071*	
C6	0.44799 (18)	0.16039 (11)	0.8243 (3)	0.0480 (6)	
C7	0.5277 (2)	0.15747 (14)	0.9552 (3)	0.0618 (7)	
H7	0.5437	0.1925	1.0180	0.074*	
C8	0.5814 (2)	0.10479 (15)	0.9913 (3)	0.0652 (8)	
H8	0.6350	0.1043	1.0757	0.078*	
C9	0.55585 (19)	0.05109 (13)	0.9008 (3)	0.0568 (7)	
H9	0.5928	0.0150	0.9252	0.068*	
C10	0.47685 (17)	0.05134 (11)	0.7766 (3)	0.0458 (5)	
H10	0.4598	0.0149	0.7204	0.055*	
C11	0.42030 (16)	0.10589 (10)	0.7312 (3)	0.0404 (5)	
C12	0.25054 (15)	0.00026 (9)	0.5662 (2)	0.0361 (4)	
C13	0.16002 (16)	0.01613 (10)	0.6138 (3)	0.0414 (5)	
C14	0.09914 (18)	−0.02789 (11)	0.6780 (3)	0.0488 (6)	
H14	0.0391	−0.0155	0.7108	0.059*	
C15	0.12767 (18)	−0.08802 (12)	0.6921 (3)	0.0501 (6)	
H15	0.0871	−0.1164	0.7350	0.060*	
C16	0.21813 (17)	−0.10821 (10)	0.6425 (3)	0.0428 (5)	
C17	0.2476 (2)	−0.17108 (11)	0.6544 (3)	0.0579 (7)	
H17	0.2070	−0.1995	0.6972	0.070*	
C18	0.3338 (2)	−0.19076 (12)	0.6049 (4)	0.0650 (8)	
H18	0.3524	−0.2322	0.6154	0.078*	
C19	0.3948 (2)	−0.14842 (12)	0.5376 (4)	0.0596 (7)	
H19	0.4534	−0.1620	0.5022	0.072*	
C20	0.36867 (17)	−0.08722 (11)	0.5238 (3)	0.0464 (5)	
H20	0.4100	−0.0601	0.4781	0.056*	
C21	0.28040 (16)	−0.06379 (10)	0.5769 (2)	0.0370 (5)	
C22	0.27474 (17)	0.06136 (10)	0.3198 (3)	0.0399 (5)	
C23	0.18677 (18)	0.04016 (11)	0.2295 (3)	0.0476 (5)	
H23	0.1395	0.0154	0.2693	0.057*	
C24	0.1777 (2)	0.06118 (15)	0.0666 (3)	0.0673 (8)	
H24	0.1230	0.0516	−0.0113	0.081*	
C25	0.2557 (2)	0.09609 (14)	0.0369 (3)	0.0647 (7)	
H25	0.2609	0.1133	−0.0628	0.078*	
C26	0.8122 (2)	0.13551 (18)	0.8359 (4)	0.0821 (10)	
H26B	0.7999	0.0928	0.8580	0.123*	
H26C	0.7565	0.1516	0.7623	0.123*	
H26D	0.8195	0.1587	0.9343	0.123*	
C27	0.9068 (2)	0.14072 (14)	0.7629 (3)	0.0598 (7)	
C28	1.0195 (3)	0.20912 (15)	0.6570 (5)	0.0830 (10)	
H28B	1.0131	0.1888	0.5531	0.100*	
H28C	1.0775	0.1919	0.7256	0.100*	
C29	1.0330 (3)	0.27622 (15)	0.6379 (5)	0.0852 (10)	
H29B	1.0927	0.2836	0.5909	0.128*	
H29C	1.0394	0.2959	0.7412	0.128*	
H29D	0.9756	0.2928	0.5691	0.128*	
O1	0.20776 (13)	0.17382 (8)	0.4507 (2)	0.0556 (4)	
H1A	0.1819	0.1373	0.4077	0.106 (13)*	
O2	0.12727 (13)	0.07581 (8)	0.5945 (2)	0.0564 (5)	
H2A	0.0650	0.0833	0.6207	0.110 (13)*	
O3	0.95846 (15)	0.09760 (9)	0.7375 (3)	0.0736 (6)	
O4	0.92817 (15)	0.19908 (10)	0.7291 (3)	0.0728 (6)	
S1	0.34342 (5)	0.10506 (3)	0.20365 (8)	0.0582 (2)	

### Atomic displacement parameters (Å^2^)

**Table:**

	*U*^11^	*U*^22^	*U*^33^	*U*^12^	*U*^13^	*U*^23^
C1	0.0354 (10)	0.0330 (10)	0.0441 (11)	−0.0001 (8)	0.0099 (9)	0.0002 (9)
C2	0.0368 (11)	0.0338 (10)	0.0439 (11)	−0.0041 (9)	0.0115 (9)	−0.0015 (9)
C3	0.0457 (12)	0.0382 (12)	0.0525 (13)	−0.0019 (10)	0.0114 (11)	−0.0021 (10)
C4	0.0652 (16)	0.0334 (12)	0.0756 (18)	−0.0004 (11)	0.0181 (14)	−0.0056 (12)
C5	0.0699 (17)	0.0439 (14)	0.0657 (17)	−0.0144 (13)	0.0177 (14)	−0.0156 (12)
C6	0.0495 (13)	0.0497 (14)	0.0467 (13)	−0.0158 (11)	0.0139 (11)	−0.0050 (10)
C7	0.0661 (17)	0.0686 (18)	0.0509 (15)	−0.0277 (15)	0.0096 (13)	−0.0073 (13)
C8	0.0548 (16)	0.089 (2)	0.0490 (15)	−0.0225 (16)	−0.0012 (12)	0.0039 (15)
C9	0.0466 (14)	0.0697 (17)	0.0529 (15)	−0.0025 (13)	0.0039 (11)	0.0125 (13)
C10	0.0427 (12)	0.0473 (13)	0.0485 (13)	−0.0057 (10)	0.0105 (10)	0.0026 (10)
C11	0.0379 (11)	0.0434 (12)	0.0424 (11)	−0.0086 (9)	0.0139 (9)	0.0000 (9)
C12	0.0358 (10)	0.0355 (11)	0.0378 (10)	−0.0043 (9)	0.0079 (9)	0.0002 (8)
C13	0.0385 (11)	0.0391 (11)	0.0472 (12)	−0.0004 (9)	0.0084 (9)	−0.0015 (9)
C14	0.0410 (12)	0.0518 (14)	0.0574 (14)	−0.0047 (10)	0.0194 (11)	−0.0018 (11)
C15	0.0462 (13)	0.0488 (13)	0.0575 (14)	−0.0121 (11)	0.0154 (11)	0.0029 (11)
C16	0.0429 (12)	0.0394 (12)	0.0461 (12)	−0.0058 (10)	0.0064 (10)	0.0028 (9)
C17	0.0620 (16)	0.0381 (12)	0.0753 (18)	−0.0091 (12)	0.0155 (14)	0.0069 (12)
C18	0.0685 (18)	0.0359 (13)	0.093 (2)	0.0037 (12)	0.0205 (16)	0.0043 (13)
C19	0.0568 (15)	0.0453 (14)	0.0800 (19)	0.0068 (12)	0.0210 (14)	−0.0009 (13)
C20	0.0437 (12)	0.0396 (12)	0.0577 (14)	0.0004 (10)	0.0132 (11)	0.0014 (10)
C21	0.0366 (10)	0.0363 (10)	0.0374 (11)	−0.0032 (9)	0.0035 (9)	0.0003 (9)
C22	0.0438 (12)	0.0347 (10)	0.0433 (12)	−0.0017 (9)	0.0131 (9)	−0.0018 (9)
C23	0.0494 (13)	0.0544 (14)	0.0393 (12)	−0.0096 (11)	0.0078 (10)	−0.0001 (10)
C24	0.0680 (18)	0.086 (2)	0.0461 (14)	−0.0053 (16)	0.0021 (13)	−0.0025 (14)
C25	0.081 (2)	0.0704 (18)	0.0453 (14)	0.0063 (15)	0.0185 (14)	0.0104 (13)
C26	0.0555 (17)	0.102 (3)	0.093 (2)	0.0005 (17)	0.0272 (16)	−0.024 (2)
C27	0.0524 (15)	0.0723 (19)	0.0556 (16)	0.0040 (14)	0.0108 (12)	−0.0122 (14)
C28	0.086 (2)	0.072 (2)	0.101 (3)	0.0128 (18)	0.047 (2)	0.0006 (18)
C29	0.084 (2)	0.075 (2)	0.097 (3)	0.0114 (18)	0.015 (2)	−0.0006 (19)
O1	0.0534 (10)	0.0441 (10)	0.0672 (11)	0.0089 (8)	0.0028 (9)	−0.0003 (8)
O2	0.0460 (9)	0.0431 (9)	0.0851 (13)	0.0081 (7)	0.0265 (9)	0.0043 (9)
O3	0.0633 (12)	0.0669 (13)	0.0975 (16)	0.0098 (10)	0.0340 (12)	−0.0031 (11)
O4	0.0678 (13)	0.0683 (13)	0.0873 (15)	0.0129 (10)	0.0281 (11)	−0.0074 (11)
S1	0.0638 (4)	0.0544 (4)	0.0600 (4)	−0.0099 (3)	0.0212 (3)	0.0090 (3)

### Geometric parameters (Å, °)

**Table:**

C1—C22	1.522 (3)	C17—C18	1.357 (4)
C1—C12	1.536 (3)	C17—H17	0.9300
C1—C2	1.548 (3)	C18—C19	1.404 (4)
C1—H1	0.9800	C18—H18	0.9300
C2—C3	1.387 (3)	C19—C20	1.369 (3)
C2—C11	1.439 (3)	C19—H19	0.9300
C3—O1	1.371 (3)	C20—C21	1.422 (3)
C3—C4	1.413 (3)	C20—H20	0.9300
C4—C5	1.357 (4)	C22—C23	1.381 (3)
C4—H4	0.9300	C22—S1	1.725 (2)
C5—C6	1.405 (4)	C23—C24	1.431 (4)
C5—H5	0.9300	C23—H23	0.9300
C6—C7	1.415 (4)	C24—C25	1.344 (4)
C6—C11	1.432 (3)	C24—H24	0.9300
C7—C8	1.357 (4)	C25—S1	1.699 (3)
C7—H7	0.9300	C25—H25	0.9300
C8—C9	1.402 (4)	C26—C27	1.497 (4)
C8—H8	0.9300	C26—H26B	0.9600
C9—C10	1.370 (3)	C26—H26C	0.9600
C9—H9	0.9300	C26—H26D	0.9600
C10—C11	1.422 (3)	C27—O3	1.201 (3)
C10—H10	0.9300	C27—O4	1.334 (4)
C12—C13	1.380 (3)	C28—O4	1.465 (4)
C12—C21	1.440 (3)	C28—C29	1.473 (4)
C13—O2	1.364 (3)	C28—H28B	0.9700
C13—C14	1.414 (3)	C28—H28C	0.9700
C14—C15	1.355 (3)	C29—H29B	0.9600
C14—H14	0.9300	C29—H29C	0.9600
C15—C16	1.412 (3)	C29—H29D	0.9600
C15—H15	0.9300	O1—H1A	0.9136
C16—C17	1.415 (3)	O2—H2A	0.9116
C16—C21	1.437 (3)		
			
C22—C1—C12	111.05 (17)	C18—C17—H17	119.3
C22—C1—C2	114.47 (17)	C16—C17—H17	119.3
C12—C1—C2	115.52 (17)	C17—C18—C19	119.8 (2)
C22—C1—H1	104.8	C17—C18—H18	120.1
C12—C1—H1	104.8	C19—C18—H18	120.1
C2—C1—H1	104.8	C20—C19—C18	120.4 (2)
C3—C2—C11	117.4 (2)	C20—C19—H19	119.8
C3—C2—C1	124.3 (2)	C18—C19—H19	119.8
C11—C2—C1	118.19 (18)	C19—C20—C21	122.2 (2)
O1—C3—C2	125.0 (2)	C19—C20—H20	118.9
O1—C3—C4	112.9 (2)	C21—C20—H20	118.9
C2—C3—C4	122.1 (2)	C20—C21—C16	116.4 (2)
C5—C4—C3	120.3 (2)	C20—C21—C12	124.04 (19)
C5—C4—H4	119.9	C16—C21—C12	119.56 (19)
C3—C4—H4	119.9	C23—C22—C1	128.5 (2)
C4—C5—C6	121.0 (2)	C23—C22—S1	110.84 (17)
C4—C5—H5	119.5	C1—C22—S1	120.59 (16)
C6—C5—H5	119.5	C22—C23—C24	111.2 (2)
C5—C6—C7	121.4 (2)	C22—C23—H23	124.4
C5—C6—C11	119.2 (2)	C24—C23—H23	124.4
C7—C6—C11	119.4 (2)	C25—C24—C23	113.7 (3)
C8—C7—C6	121.6 (3)	C25—C24—H24	123.2
C8—C7—H7	119.2	C23—C24—H24	123.2
C6—C7—H7	119.2	C24—C25—S1	111.8 (2)
C7—C8—C9	119.7 (3)	C24—C25—H25	124.1
C7—C8—H8	120.1	S1—C25—H25	124.1
C9—C8—H8	120.1	C27—C26—H26B	109.5
C10—C9—C8	120.5 (3)	C27—C26—H26C	109.5
C10—C9—H9	119.7	H26B—C26—H26C	109.5
C8—C9—H9	119.7	C27—C26—H26D	109.5
C9—C10—C11	121.8 (2)	H26B—C26—H26D	109.5
C9—C10—H10	119.1	H26C—C26—H26D	109.5
C11—C10—H10	119.1	O3—C27—O4	123.1 (3)
C10—C11—C6	116.9 (2)	O3—C27—C26	124.4 (3)
C10—C11—C2	123.2 (2)	O4—C27—C26	112.5 (3)
C6—C11—C2	119.9 (2)	O4—C28—C29	108.4 (3)
C13—C12—C21	118.02 (18)	O4—C28—H28B	110.0
C13—C12—C1	120.75 (18)	C29—C28—H28B	110.0
C21—C12—C1	121.17 (18)	O4—C28—H28C	110.0
O2—C13—C12	118.84 (19)	C29—C28—H28C	110.0
O2—C13—C14	119.1 (2)	H28B—C28—H28C	108.4
C12—C13—C14	122.0 (2)	C28—C29—H29B	109.5
C15—C14—C13	120.4 (2)	C28—C29—H29C	109.5
C15—C14—H14	119.8	H29B—C29—H29C	109.5
C13—C14—H14	119.8	C28—C29—H29D	109.5
C14—C15—C16	121.0 (2)	H29B—C29—H29D	109.5
C14—C15—H15	119.5	H29C—C29—H29D	109.5
C16—C15—H15	119.5	C3—O1—H1A	111.5
C15—C16—C17	121.3 (2)	C13—O2—H2A	115.5
C15—C16—C21	119.0 (2)	C27—O4—C28	116.7 (2)
C17—C16—C21	119.7 (2)	C25—S1—C22	92.43 (13)
C18—C17—C16	121.4 (2)		

### Hydrogen-bond geometry (Å, °)

**Table:**

*D*—H···*A*	*D*—H	H···*A*	*D*···*A*	*D*—H···*A*
O2—H2A···O3^i^	0.91	1.88	2.764 (3)	163

Symmetry codes: (i) *x*−1, *y*, *z*.
